# Trend analysis and survival of primary gallbladder cancer in the United States: a 1973–2009 population‐based study

**DOI:** 10.1002/cam4.1044

**Published:** 2017-03-20

**Authors:** Rubayat Rahman, Eduardo J. Simoes, Chester Schmaltz, Christian S. Jackson, Jamal A. Ibdah

**Affiliations:** ^1^ Division of Gastroenterology and HepatologyUniversity of Missouri School of MedicineOne Hospital Drive, CE 405ColumbiaMissouri65212; ^2^ Department of Health Management and InformaticsUniversity of Missouri School of MedicineOne Hospital DriveCE707 CS&E BldgColumbiaMissouri65212; ^3^ Missouri Cancer Registry and Research CenterUniversity of Missouri at Columbia401 Clark HallColumbiaMissouri65211; ^4^ Section of GastroenterologyLoma Linda VA Medical CenterLoma LindaCalifornia

**Keywords:** Epidemiology, gallbladder cancer, incidence trend, SEER, survival trend

## Abstract

Primary gallbladder cancer is an aggressive and uncommon cancer with poor outcomes. Our study examines epidemiology, trend, and survival of gallbladder cancer in the United States from 1973 to 2009. We utilized the Surveillance Epidemiology and End Results database (SEER). Frequency and rate analyses on demographics, stage, and survival were compared among non‐Hispanic whites, Hispanics, African American, and Asian/Pacific Islanders. A total of 18,124 cases were reported in SEER from 1973 to 2009 comprising 1.4% of all reported gastrointestinal cancers. Gallbladder cancer was more common in females than males (71 vs. 29%, respectively). The age‐adjusted incidence rate was 1.4 per 100,000, significantly higher in females than males (1.7 vs. 1.0). Trend analysis showed that the incidence rate has been decreasing over the last three decades for males. However, among females, the incidence rate had decreased from 1973 to mid‐90s but has remained stable since then. Trend analysis for stage at diagnosis showed that the proportion of late‐stage cases has been increasing significantly since 2001 after a decreasing pattern since 1973. Survival has improved considerably over time, and survival is better in females than males and in Asian/Pacific Islanders than other racial groups. The highest survival was in patients who received both surgery and radiation. Trend analysis revealed a recent increase of the incidence of late‐stage gallbladder cancer. Highest survival was associated with receiving both surgery and radiation.

## Introduction

Primary gallbladder cancer (GBC) is a rare gastrointestinal malignancy but is the most common cancer arising in the biliary tract, representing 80–95% of all biliary tract cancers worldwide 1, 2. Gallbladder cancer remains an aggressive cancer with an overall dismal outcome despite all the biomedical, technological, surgical, and chemotherapeutic advancements in the last decade. It is estimated that <5000 new cases are diagnosed each year in the US. Based on the available data, the incidence of GBC is 1–2 cases per 100,000 population in the US 2, 3. Incidence steadily increases with age in both sexes, women are affected 2–6 times more often than men, and GBC is more common in non‐Hispanic whites (NHW) than in African Americans (AA) 2, 4. Several risk factors have been identified for GBC including gallstone disease, gallbladder polyps, chronic infection (Salmonella, Helicobacter), congenital biliary cyst, abnormal pancreaticobiliary duct junction, carcinogen exposure, obesity, and diabetes mellitus 5, 6.

Most of the GBC cases are found incidentally in patients undergoing either laparoscopic or open exploration for cholelithiasis and/or cholecystitis. It is estimated that GBC can be found in 2% of cholecystectomies 7. About 70% of cases of GBC are diagnosed at the regional or distant stage. Currently, the role of adjuvant radiation therapy in GBC treatment is not well established. Retrospective series have suggested some degree of benefit from adjuvant chemotherapy in the treatment of GBC but prospective trials have been limited by the lack of effective agents in this regard. However, even after complete resection, outcomes are poor, particularly for lesions penetrating through the serosa, invading liver or adjacent structures or node‐positive disease. If GBC is resectable at the time of diagnosis, then resection is followed by adjuvant chemotherapy with or without radiation. For unresectable cases, chemotherapy is the modality of treatment 8, 9. Observed survival rates from a series of 10,705 cases of GBC collected between 1989 and 1996 in the National Cancer Database and stratified according to stage at diagnosis using the newest American Joint Committee on Cancer (AJCC) criteria showed 50% and 2% survival at 5‐years for stage I and IV, respectively 10. Overall poor prognosis and survival associated with GBC is postulated to be related to an advanced stage at diagnosis, which is due both to the anatomic position of the gallbladder and the nonspecificity of symptoms 11. With the introduction of laparoscopic cholecystectomy, greater numbers of cholecystectomies have been performed. It has been postulated that this change may have contributed to earlier incidental diagnosis of GBC in all stages with some improvement in overall survival 12.

Gallbladder cancer has been understudied. Only few studies have been published in the last decade on epidemiology and survival of GBC in the United States 13, 14, 15. Most of the published studies focused on the surgical management of GBC and its associated survival 7, 8, 9, 16. With all the recent improvement of GBC therapeutic modalities, there is a great need to explore the above mentioned factors at the population level. The goal of our study is to examine demographics and trends using a large population data source, Surveillance, Epidemiology and End Results (SEER) database, from 1973 to 2009, and to compare the treatment modalities of surgery and radiation on survival.

## Methods

### Data source

SEER, a program of National Cancer Institute (NCI) is a source of population‐based cancer surveillance information in the US. SEER collects information on incidence, prevalence, and survival from specific geographic areas representing 28% of the total US population in 2010. The SEER Cancer Incidence Research Database consists of tumors reported to 18 registries since 2000, 13 registries since 1992, and nine registries since 1973. Geographic areas were selected for inclusion in the SEER program based on their ability to operate and maintain a high‐quality population‐based cancer reporting system and for their epidemiologically significant population subgroups. The population covered by SEER is comparable to the general US population with regard to measures of poverty and education. The SEER population tends to have a higher proportion of foreign‐born persons than the general US population 17.

### Study population

From 1973, SEER used the following broad racial categories: white, African American, American Indian/Alaska Native, Asian/Pacific Islander, and other. Hispanics were identified from the NAACCR Hispanic Identification Algorithm (NHIA) (Hispanics and non‐Hispanics) variable. Our study population included patients diagnosed with GBC residing in SEER registries areas and reported in SEER database from 1973 to 2009 17. The analyses were performed according to major US race/ethnic groups—non‐Hispanic whites (NHW), Hispanics (excluding those reported by the Alaska Native Registry), African Americans (AA), and Asians and Pacific Islanders (A/PI).

### Statistical analyses

We used SEER*Stat and SAS software for the descriptive analyses 17, 18. Joinpoint Regression Program was used to analyze trends in rates and distribution of stage. We calculated the overall and gender‐specific incidence rate of GBC adjusted for age based on US Census Data 2000. We implemented trend analysis of GBC age‐adjusted rates from 1973 to 2009 according to gender, and from 1992 to 2009 by race/ethnicity. Rates by race/ethnicity were limited to 1992–2009 due to the unavailability of a population file in the SEER database with detailed race/ethnicity for the years 1973–1991. We used SEER Historic Summary Stage data to generate trend analysis for stage at diagnosis from 1973 to 2009.

SAS was used to compare the distribution of GBC cases by descriptive variables across race/ethnicity groups 18. We used Cox proportional hazard regression in SAS to calculate the adjusted relative hazard (RH) of GBC‐related death. The survival analysis was performed with three endpoints: any cause of death, GBC‐specific cause of death, and other cause of death. The last two endpoints are based on the variables SEER Cause‐Specific Death Classification and SEER Other Cause of Death Classification.

The proportional hazard model's main independent variables included whether the patient had surgery or radiation. Additional covariates included age in 5‐year groups, year of diagnosis in 5‐year periods, race in four groups, marital status, sex, histology in nine groups, stage and grade. Missing values were assumed to be missing at random and those cases were simply dropped from the analysis. A two‐tailed *P* value of 0.05 or less was considered statistically significant.

## Results

### Demographics

A total of 18,124 incident cases of GBC were reported in SEER database from 1973 to 2009. It comprises about 1.4% of all gastrointestinal malignancies reported in the database during this time period. Table 1 shows the demographics of reported GBC in SEER database from 1973 to 2009. Gallbladder cancer is significantly (*P* < 0.0001) more common in females (71%) than males (29%). Most of the cases were reported in NHW (66%) followed by Hispanics (16%), while 9% of cases were reported in AA and 8% cases in A/PI. Compared to NHW (71%), more Hispanics females (78%) and less A/PI females (63%) were diagnosed with GBC (*P* < 0.0001).

**Table 1:** Descriptive statistics of gallbladder cancer (GBC) in different race/ethnicities as reported in SEER database 1973–2009

	All races	NHW (referent group)	Tested against NHW		
			AA	A/PI	Hispanic
	*N* = 18,124 (100%)	*N* = 11,897 (66%)	*N* = 1547 (9%)	*N* = 1484 (8%)	*N* = 2899 (16%)
% male (95% CI)	29 (28, 29)	29 (28, 30)	30 (28, 33)	37^a^ (35, 40)	22^a^ (20, 23)
% age <65 (95% CI)	27 (27, 28)	22 (21, 23)	39^a^ (36, 41)	29^a^ (27, 32)	40^a^ (38, 42)
% age 65–79 (95% CI)	45 (44, 45)	45 (45, 46)	42^a^ (40, 44)	46 (44, 49)	42^a^ (40, 44)
% age 80+ (95% CI)	28 (28, 29)	32 (32, 33)	20^a^ (18, 22)	25^a^ (23, 27)	18^a^ (17, 19)
% diagnosed 2000–2009 (95% CI)	50 (50, 51)	46 (45, 47)	63^a^ (60, 65)	55^a^ (52, 57)	62^a^ (60, 64)
% married (95% CI)	49 (48, 49)	49 (48, 50)	38^a^ (36, 41)	60^a^ (57, 63)	47^a^ (45, 48)
% localized stage (95% CI)	31 (30, 32)	30 (29, 31)	31 (29, 34)	33^a^ (31, 36)	33^a^ (31, 35)
% low grade (grade I and II) (95% CI)	53 (52, 54)	52 (51, 53)	51 (48, 54)	57^a^ (54, 60)	52 (50, 54)
% no surgery (95% CI)	34 (33, 34)	33 (33, 34)	35 (33, 38)	35 (38, 54)	33 (31, 34)
% no radiation (95% CI)	87 (86, 87)	87 (86, 87)	86 (84, 88)	85^a^ (83, 86)	87 (86, 88)

NHW, non‐hispanic white, AA, African American, A/PI, Asian and Pacific Islander.

a *P* < 0.05 in comparison to referent group.

### Incidence rate and trend

**Figure 1:**
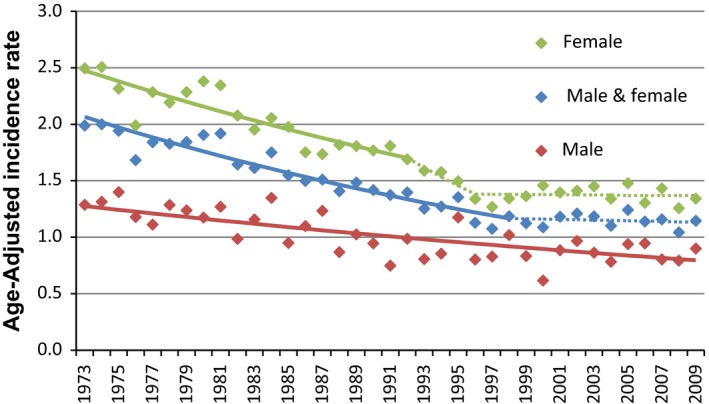
Trend of Incidence rate of gallbladder cancer. As reported in Surveillance Epidemiology and End Results database 1973–2009. Segments calculated by the Joinpoint Regression program on the log‐scale, dotted if the APC differs insignificantly from zero.

The overall age‐adjusted incidence rate of GBC for the study period of 1973–2009 is 1.4 per 100,000. It is higher in females than males (1.7 vs. 1.0 per 100,000; *P* < 0.0001). In the SEER‐13 from 1992 to 2009, the rates are significantly higher for AA, A/PI, and Hispanics than NHW for both males and females. Moreover, the rate for females is higher than males across each of these four race/ethnic groups. Trend analysis showed that the incidence rates for both males and females have generally decreased significantly since 1973 (Fig. 1) as reported in SEER database with the rates for females having leveled‐off since the mid‐’90s.

### Stage at diagnosis and trend

**Figure 2:**
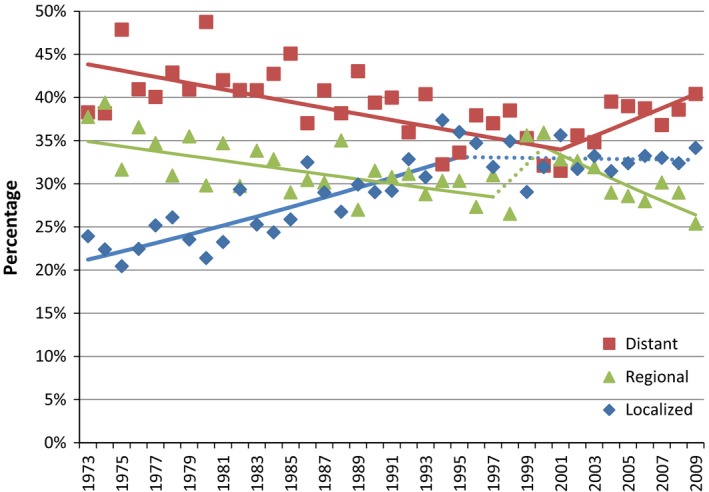
Trend of gallbladder cancer stages at diagnosis. As reported in Surveillance Epidemiology and End Results database 1973–2009. Segments calculated by the Joint point Regression Program on the logit‐scale. Solid lines indicate significant trends. Dotted lines indicate insignificant trends (APC differs insignificantly from zero).

Trend analysis shows that the proportion of cases diagnosed at localized stage increased significantly since 1973, but appears to have stabilized since around the mid‐’90s (Fig. 2). The proportion of cases diagnosed at regional stage has been generally decreasing since 1973 with a possible jump occurring in the mid to late ‘90s. Unfortunately, the proportion of distant stage cases has been increasing significantly since around the beginning of this century after a decreasing pattern since 1973. The distribution of stage was calculated for three time periods that roughly correspond to the two joint points seen in the proportion of localized and distant stage. The AAPC over 2000–2009 is significantly <0 for regional, significantly greater than zero for distant, and differs insignificantly from zero for localized. In the most recent time period 2000–2009, the percent of cases that are regional is significantly less than that of localized, and the percent of cases that are distant stage are significantly higher than that of localized. A/PIs and Hispanics have a higher portion of cases diagnosed at the localized stage over 1973–2009 (Table 1).

### Survival analysis

A total of 15,513 cases (86%) were selected for inclusion in survival analysis. Only patients who were diagnosed with GBC as their first reportable malignant primary tumor were included in survival analysis. Table 2 shows the variables that affected the relative hazard of death (RH). RH of a GBC‐related death as well as for any cause of death was significantly higher with older age, later stages, and higher grades at diagnosis. Survival is also better in females than males and in Asian/Pacific Islanders than other racial groups. The patients who did not receive any surgery had higher RHs for GBC‐specific and any cause of death in comparison to patients who received surgery as either the only modality of treatment or in combination with radiation therapy. Among patients who did not receive radiation, not receiving surgery was also associated with a higher hazard of a non‐GBC‐specific cause of death. Similarly, not receiving radiation was associated with higher relative hazard for all three end‐points. The highest survival was in patients who received both types of treatment (Table 2).

**Table 2:** Relative hazards of death in gallbladder cancer (GBC) cases according to various characteristics as reported in SEER database 1973–2009

Variables	Any cause of death	GBC‐specific cause of death	Other cause of death
No surgery/no radiation (95% CI)	1.8^a^ (1.7, 1.9)	1.8^a^ (1.7, 1.9)	1.5^a^ (1.3, 1.8)
No surgery/with radiation (95% CI)	1.6^a^ (1.4, 1.8)	1.6^a^ (1.4, 1.8)	0.8 (0.5, 1.4)
Surgery	1 (Ref.)	1 (Ref.)	1 (Ref.)
No radiation/no surgery (95% CI)	1.6^a^ (1.4, 1.8)	1.5^a^ (1.4, 1.7)	2.9^a^ (1.7, 4.9)
No radiation/with surgery (95% CI)	1.4^a^ (1.3, 1.5)	1.4^a^ (1.3, 1.5)	1.6^a^ (1.3, 1.9)
Radiation	1 (Ref.)	1 (Ref.)	1 (Ref.)
Age 10–34	1 (Ref.)	1 (Ref.)	1 (Ref.)
Age 35–39 (95% CI)	1.4 (0.9, 2.0)	1.3 (0.9, 2.0)	1.4 (0.5, 3.8)
Age 40–44 (95% CI)	1.6^a^ (1.1, 2.3)	1.5^a^ (1.0, 2.2)	1.4 (0.5, 3.7)
Age 45–49 (95% CI)	1.8^a^ (1.3, 2.6)	1.7^a^ (1.2, 2.5)	1.9 (0.7, 4.8)
Age 50–54 (95% CI)	1.9^a^ (1.3, 2.6)	1.8^a^ (1.3, 2.6)	1.4 (0.6, 3.6)
Age 55–59 (95% CI)	1.9^a^ (1.4, 2.7)	1.8^a^ (1.2, 2.5)	2.2 (0.9, 5.4)
Age 60–64 (95% CI)	2.3^a^ (1.6, 3.1)	2.0^a^ (1.4, 2.9)	2.9^a^ (1.2, 7.1)
Age 65–69 (95% CI)	2.4^a^ (1.7, 3.3)	2.1^a^ (1.5, 3.0)	3.4^a^ (1.4, 8.4)
Age 70–74 (95% CI)	2.6^a^ (1.9, 3.6)	2.2^a^ (1.6, 3.2)	4.4^a^ (1.8, 10.8)
Age 75–79 (95% CI)	3.2^a^ (2.3, 4.4)	2.7^a^ (1.9, 3.9)	5.3^a^ (2.1, 12.8)
Age 80–84 (95% CI)	3.5^a^ (2.6, 4.9)	2.9^a^ (2.0, 4.1)	8.0^a^ (3.3, 19.4)
Age 85+ (95% CI)	4.6^a^ (3.3, 6.3)	3.6^a^ (2.5, 5.1)	11.4^a^ (4.7, 27.7)
Diagnosed 1973–1979 (95% CI)	1.6^a^ (1.5, 1.7)	1.7^a^ (1.6, 1.8)	1.1 (0.9, 1.4)
Diagnosed 1980–1984 (95% CI)	1.5^a^ (1.4, 1.6)	1.6^a^ (1.4, 1.7)	1.1 (0.9, 1.4)
Diagnosed 1985–1989 (95% CI)	1.4^a^ (1.3, 1.5)	1.4^a^ (1.3, 1.5)	1.1 (0.9, 1.4)
Diagnosed 1990–1994 (95% CI)	1.3^a^ (1.2, 1.4)	1.4^a^ (1.3, 1.5)	1.1 (0.9, 1.3)
Diagnosed 1995–1999 (95% CI)	1.2^a^ (1.1, 1.3)	1.2^a^ (1.1, 1.3)	1.2 (1.0, 1.4)
Diagnosed 2000–2004 (95% CI)	1.1^a^ (1.1, 1.2)	1.2^a^ (1.1, 1.2)	0.9 (0.8, 1.1)
Diagnosed 2005–2009	1 (Ref.)	1 (Ref.)	1 (Ref.)
Non‐Hispanic White	1 (Ref.)	1 (Ref.)	1 (Ref.)
African American (95% CI)	1.1^a^ (1.0, 1.2)	1.0 (1.0, 1.1)	1.4^a^ (1.2, 1.7)
American Indian/Alaska Native (95% CI)	1.2^a^ (1.1, 1.4)	1.2^a^ (1.0, 1.4)	1.3 (0.9, 1.9)
Asian or Pacific Islander (95% CI)	0.9^a^ (0.8, 0.9)	0.8^a^ (0.8, 0.9)	0.9 (0.8, 1.1)
Not Hispanic	1 (Ref.)	1 (Ref.)	1 (Ref.)
Hispanic (95% CI)	1.0 (0.9, 1.0)	1.0 (0.9, 1.0)	1.1 (1.0, 1.3)
Localized stage	1 (Ref.)	1 (Ref.)	1 (Ref.)
Regional stage (95% CI)	2.4^a^ (2.3, 2.5)	2.9^a^ (2.8, 3.1)	1.2^a^ (1.0, 1.3)
Distant stage (95% CI)	3.8^a^ (3.6, 4.0)	4.8^a^ (4.5, 5.1)	1.2 (1.0, 1.4)
Well differentiated (grade I)	1 (Ref.)	1 (Ref.)	1 (Ref.)
Moderately differentiated (grade II) (95% CI)	1.2^a^ (1.2, 1.3)	1.3^a^ (1.2, 1.5)	1.0 (0.9, 1.2)
Poorly differentiated (grade III) (95% CI)	1.7^a^ (1.5, 1.8)	1.8^a^ (1.7, 2.0)	1.2^a^ (1.1, 1.4)
Undifferentiated, anaplastic (grade IV) (95% CI)	1.7^a^ (1.5, 2.0)	1.9^a^ (1.7, 2.2)	1.1 (0.8, 1.6)
Married	1 (Ref.)	1 (Ref.)	1 (Ref.)
Separated or not married (95% CI)	1.1^a^ (1.0, 1.1)	1.1^a^ (1.0, 1.1)	1.3^a^ (1.2, 1.4)
Male (95% CI)	1.1^a^ (1.1, 1.2)	1.1^a^ (1.0, 1.1)	1.5^a^ (1.3, 1.6)
Female	1 (Ref.)	1 (Ref.)	1 (Ref.)
Unspecified neoplasms (95% CI)	1.2^a^ (1.1, 1.4)	1.2^a^ (1.0, 1.4)	1.4^a^ (1.0, 2.0)
Epithelial neoplasms, NOS (95% CI)	1.0 (1.0, 1.1)	1.0 (1.0, 1.1)	1.0 (0.9, 1.3)
Squamous cell neoplasm (95% CI)	1.2^a^ (1.1, 1.4)	1.2^a^ (1.0, 1.4)	1.2 (0.9, 1.7)
Adenomas and adenocarcinomas	1 (Ref.)	1 (Ref.)	1 (Ref.)
Cystic, mucinous, and serious neoplasm (95% CI)	1.1^a^ (1.1, 1.2)	1.2^a^ (1.1, 1.3)	0.9 (0.7, 1.1)
Ductal neoplasm (95% CI)	1.3 (0.9, 1.7)	1.3 (0.9, 1.7)	1.1 (0.4, 3.5)
Complex epithelial neoplasms (95% CI)	1.4^a^ (1.2, 1.5)	1.4^a^ (1.3, 1.6)	0.9 (0.6, 1.3)
Complex mixed and stromal neoplasm (95% CI)	1.1 (0.8, 1.5)	1.3 (0.9, 1.8)	0.2 (0.03, 1.3)
Other histologic groups (95% CI)	1.1 (0.7, 1.7)	0.9 (0.6, 1.5)	2.7^a^ (0.2, 6.1)

a *P *<* *0.05 in comparison to referent group.

## Discussion

Gallbladder cancer is an uncommon malignancy of the hepatobiliary tract. Currently GBC ranks fifth among gastrointestinal cancers. The global rates for GBC exhibit significant variability, reaching epidemic levels for some specific geographic regions and ethnicities. The basis for this wide variance most likely resides in differences in environmental exposures, incidence, and prevalence of risk factors for GBC 2. Nevertheless, its high fatality rate continues to pose a significant dismal outcome. With the changing risk exposure and stratification, specially increasing obesity, cholelithiasis, and increasing surgical interventions for many gallbladder diseases, the epidemiology and survival of GBC is changing. Extended surgical resection, lymph node excision, or both may improve survival in certain patients with incidentally discovered gallbladder cancer 13, 14, 18. To date, this study is the largest population‐based study to evaluate the epidemiology, trend, and survival of GBC in the United States.

During the study period, incidence rate of GBC remained low in comparison to other gastrointestinal or hepatobiliary malignancies. The global historic data also suggest that it remained low in many other parts of the world 19. The incidence rate has been decreasing over the last three decades for males. However, among females, the incidence rate had decreased from 1973 to the mid‐’90s but has remained stable since then. During the study period, the proportion of localized stage GBC has increased until the mid‐’90s; this shift may be explained by increased incidental findings of GBC at cholecystectomy, as well as improved treatment modalities for many gallbladder pathologies. This was noted among all races and it is reasonable to conclude that detection at early stages is a major contributor for increased GBC survival 20, 21. However, our trend analysis reveals an alarming observation: there is a recent significant surge in the proportion of late stages at diagnosis since 2001 after a decline over long period of time since 1973. This alarming change in the trend deserves further evaluation and study. It is not documented in the available literature whether changes in risk factors or diagnostic modalities account for such increase in late‐stage GBC.

Our study shows that survival of GBC is improving significantly over time. Survival is better in females than males and in A/PI than other groups. Patients who did not receive any surgery had higher RHs for GBC‐specific and any cause of death in comparison to patients who received surgery as either the only modality of treatment or in combination with radiation. Among patients who did not receive radiation, not receiving surgery was also associated with a higher hazard of a non‐GBC‐specific cause of death. Similarly, not receiving radiation was associated with higher relative hazards for all three end‐points. The highest survival was in patients who received both types of treatment. The higher hazards for non‐GBC‐related death associated with no treatment suggests that patients who did not receive treatment were at a higher risk of death overall. The higher survival in patients who underwent treatment may reflect in part the higher expectation of survival in those patients compared to those who did not undergo treatment. The information provided by SEER, however, remains limited and does not allow for further assessment of various treatment modalities on survival. Randomized controlled trials are needed to further evaluate the effects of surgery and/or radiation on survival. Nevertheless, this study provides valuable contribution to the literature by conducting a powerful analysis of the variables impacting survival, including the observed beneficial effect of surgery and radiation, in large number of cases analyzed over approximately four decades. The beneficial survival impact of surgical approach on gallbladder cancer has been mixed. However, an aggressive surgical approach for patients with gallbladder cancer has been reported to substantially and significantly improve survival of gallbladder cancer patients 22. In one study, the overall 5‐year survival for patients with GBC who underwent curative resection was 21–69% 23. In another study, 18% of patients received radiation therapy after curative intent surgery. The use of adjuvant radiation therapy was associated with a short‐term survival benefit, but the benefit dissipated over time 24. Although surgery is the mainstay of treatment for early or localized GBC, the study showed that few patients underwent aggressive surgery 25. The common characteristic of these previously published studies have been their small number of surgical cases followed up and the observational natures of their design. Our study contributes to the previous studies with large number of cases and the ability to control for many intervening factors.

In this epidemiological study, we used the comprehensive and quality‐controlled SEER database, which adds significant strength to our analysis. The SEER‐18 registries which have reportable tumors from the year 2000 and later covered more than 28% of the total US population in 2010; cases linked to population data with the major race/ethnic categories are available back to 1992 for 13 of the registries; and data on tumors diagnosed back to 1973 are available for the SEER‐9 registries with the major race/ethnic categories but with population race data only in the groups categorized as white, AA, and other. The population covered by SEER registries program is highly comparable to the general US population; however, it tends to be somewhat more urban than rural and has a higher proportion of foreign‐born persons than the general US population 17. Another limitation is SEER's lack of information on comorbidities or access to health insurance for the period studied, which can be a confounder in the survival analysis. Further, SEER database lacks comprehensive analysis of the type and extent of surgery for the period studied.

In conclusion, GBC is rare cancer of the gastrointestinal tract, but its incidence has generally decreased over the past several decades and appears to have stabilized for females. We also document a trend change in the staging pattern of GBC revealing the proportion of cases diagnosed at distant stage increasing in recent years after a long period of decline since the 1970s. Along with these findings, we have revealed a significantly improved survival of GBC over time. Highest survival was associated with receiving both surgery and radiation.

## Conflict of Interest

None declared.
